# The Role of p53 in Progression of Cutaneous Squamous Cell Carcinoma

**DOI:** 10.3390/cancers13184507

**Published:** 2021-09-07

**Authors:** Minna Piipponen, Pilvi Riihilä, Liisa Nissinen, Veli-Matti Kähäri

**Affiliations:** 1 Department of Dermatology, University of Turku and Turku University Hospital, Hämeentie 11 TE6, FI-20520 Turku, Finland; mmpiip@utu.fi (M.P.); pimati@utu.fi (P.R.); liinis@utu.fi (L.N.); 2 FICAN West Cancer Centre Research Laboratory, University of Turku and Turku University Hospital, Kiinamyllynkatu 10, FI-20520 Turku, Finland; 3 Center for Molecular Medicine, Department of Medicine Solna, Dermatology and Venereology Division, Karolinska Institute, 17176 Stockholm, Sweden

**Keywords:** p53, skin, cancer, squamous cell carcinoma

## Abstract

**Simple Summary:**

Skin cancers are the most common types of cancer worldwide, and their incidence is increasing. Epidermal keratinocyte-derived cutaneous squamous cell carcinoma (cSCC) is the most common metastatic skin cancer, and it is associated with poor prognosis in the advanced stage. The most important risk factor for cSCC is long-term exposure to solar ultraviolet radiation, which induces oncogenic mutations in epidermal keratinocytes. The most common mutations are inactivating mutations in tumor suppressor p53, which result in accumulation of additional mutations. Recently, the role of p53 in the progression and invasion of cSCC has also been elucidated. In this review we will discuss the role of p53 in development of cSCC and as a potential new therapeutic target in advanced cSCC.

**Abstract:**

Skin cancers are the most common types of cancer worldwide, and their incidence is increasing. Melanoma, basal cell carcinoma (BCC), and cutaneous squamous cell carcinoma (cSCC) are the three major types of skin cancer. Melanoma originates from melanocytes, whereas BCC and cSCC originate from epidermal keratinocytes and are therefore called keratinocyte carcinomas. Chronic exposure to ultraviolet radiation (UVR) is a common risk factor for skin cancers, but they differ with respect to oncogenic mutational profiles and alterations in cellular signaling pathways. cSCC is the most common metastatic skin cancer, and it is associated with poor prognosis in the advanced stage. An important early event in cSCC development is mutation of the *TP53* gene and inactivation of the tumor suppressor function of the tumor protein 53 gene (TP53) in epidermal keratinocytes, which then leads to accumulation of additional oncogenic mutations. Additional genomic and proteomic alterations are required for the progression of premalignant lesion, actinic keratosis, to invasive and metastatic cSCC. Recently, the role of p53 in the invasion of cSCC has also been elucidated. In this review, the role of p53 in the progression of cSCC and as potential new therapeutic target for cSCC will be discussed.

## 1. Introduction

Skin cancers are the most common types of cancer worldwide, and their incidence is increasing. Melanoma, basal cell carcinoma (BCC), and cutaneous squamous cell carcinoma (cSCC) are the three major types of skin cancer. Melanoma originates from melanocytes, whereas BCC and cSCC originate from epidermal keratinocytes. BCC is derived from basal cells of the interfollicular epidermal layer, and cSCC is derived from keratinocytes of the interfollicular epidermal layer and hair follicle stem cells [1]. Chronic exposure to ultraviolet radiation (UVR), especially to UVB, is a common risk factor for these skin cancers, but they differ with respect to oncogenic mutation profiles and alterations in cellular signaling pathways [1]. BCCs and cSCCs harbor a high burden of UV-induced mutations, but they do not share many genetic alterations, except inactivation of tumor suppressor p53. In BCC, loss of PTCH1 receptor function results in activation of the G protein-coupled receptor SMO and constitutive activation of the Hedgehog signaling pathway, whereas in cSCC, mutation and inactivation of p53 is an important early pathogenic event [1].

cSCC is the most common metastatic skin cancer [1,2]. The mutation rate of cSCC is one of highest among the malignant tumors, and the majority of mutations found in cSCC are UV-induced [1]. The important early event in cSCC development is mutation and inactivation of tumor suppressor function of the tumor protein 53 gene (TP53), which in turn leads to accumulation of additional oncogenic mutations including the loss-of-function mutation of *NOTCH1* [1]. Inactivation of p53 also results in the downregulation of *NOTCH1* expression. Furthermore, driver mutations in cSCC have been identified in different genes, i.e., *NOTCH2*, *EGFR, HRAS*, *KRAS*, and *PIK3CA*. However, these mutations are also found with high frequency in normal epidermal keratinocytes in chronically sun-exposed skin. It is therefore likely that additional genomic and proteomic alterations are required for the progression of premalignant lesion, actinic keratosis (AK), to cSCC in situ (cSCCIS), and finally to invasive and metastatic cSCC. In this review, the role of p53 in the progression of cSCC and as a potential new therapeutic target for cSCC will be discussed.

## 2. Cutaneous Squamous Cell Carcinoma

### 2.1. Epidemiology, Clinical Presentation, and Risk Factors of cSCC

Cutaneous squamous cell carcinoma (cSCC) is the most common keratinocyte-derived carcinoma with metastatic potential, and it is the second most common skin cancer after BCC [1,2,3]. The incidence of cSCC is increasing worldwide, and it is estimated that at least 20% of skin cancer-related deaths are caused by cSCC [2,4,5,6,7,8]. Approximately 2–4% of primary cSCCs metastasize, primarily to local lymph nodes, causing disease-specific mortality [2,8,9]. Moreover, in cSCC patients, the risk for other primary tumors is increased, and the overall risk of mortality is raised [10]. The risk for metastasis is higher in immunosuppressed individuals and in organ transplant recipients [1,2,4,11,12,13,14]. The prognosis of patients with metastatic cSCC is poor, with 3-year overall survival (OS) of 29–46% [7,13]. Moreover, approximately 50% of metastases are discovered within 6 months from the detection of the primary cSCC tumor [7]. To date, there are no established biomarkers in clinical practice to predict the aggressiveness, poor prognosis, or risk of recurrence of primary cSCC. Thus, there is an unmet medical need for risk assessment and new therapeutic strategies for cSCC [15].

cSCCs are derived from keratinocytes, which are exposed to sunlight on a daily basis, and cumulative exposure to solar UVR is the most important risk factor for cSCC. Therefore, cSCC and its precursor lesions are typically detected in sun-exposed skin, such as in the head and neck region [16,17,18]. Other important risk factors for cSCC include fair skin, immunosuppression, male sex, advanced age, chronic cutaneous ulceration, chronic inflammation, human papilloma virus (HPV) infection, smoking, BRAF inhibitor medication, chronic lymphocytic leukemia, non-Hodgkin lymphoma, and recessive dystrophic epidermolysis bullosa [1,2,5,19,20,21,22,23,24,25,26].

**Figure 1:**
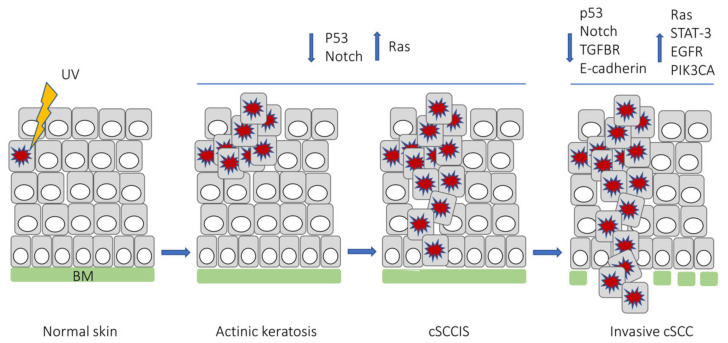
Molecular features involved in the development of actinic keratosis, cutaneous squamous cell carcinoma in situ (cSCCIS), and invasive cSCC. BM, basement membrane; TGFBR, transforming growth factor-β receptor; EGFR, epidermal growth factor receptor; PIK3CA, phosphatidylinositol-4,5-bisphosphate 3-kinase catalytic subunit α.

cSCC progresses from premalignant lesions, i.e., AK to cSCCIS, and finally to invasive cSCC (Figure 1) [1]. Clinical appearance of cSCC is variable, and it can be observed as indurated, smooth plaque, or exophytic and ulcerative lesion. The precursor lesions, AK and cSCCIS, are not always clinically distinguished from cSCC lesions. Thus, the diagnosis of primary cSCC relies on histopathological examination of lesional biopsy [2,14,27].

Only a portion of AKs will progress to invasive cSCC [1,28]. The risk for invasive cSCC increases if several AK lesions are present, and AKs are the most potent predictive factors for cSCC progression [5]. Occurrence of several concurrent AK lesions on a cSCC patient is a sign of field cancerization, and it increases the risk of second primary cSCC or nodal metastasis [29]. However, it has been shown that the AK or cSCCIS lesions detected prior to cSCC diagnosis correlate with lower metastasis risk and could function as a protective factor for cSCC metastasis [7,28]. The metastases are typically detected in locoregional lymph nodes, and distant metastases are less common [4]. The tumor-associated risk factors for metastases in primary cSCC are location on temple, tumor diameter over 20 mm, tumor invasion beyond subcutaneous fat, rapid tumor growth, previous recurrence of cSCC tumor, presence of several cSCC tumors, and neurological symptoms (paraesthesia and pain) [30,31,32,33,34]. Histological risk factors for local recurrence and metastasis are invasion depth, perineural or lympho-vascular invasion, poor differentiation grade, and specific histologic subtypes such as acantholytic, adenosquamous, sarcomatoid, or desmoplastic cSCC [27,29]. Furthermore, cSCCs developing in certain sun-protected skin areas, such as soles of the feet or perineum, are associated with higher metastasis risk [35].

### 2.2. Molecular Alterations in Pathogenesis of cSCC

**Figure 2:**
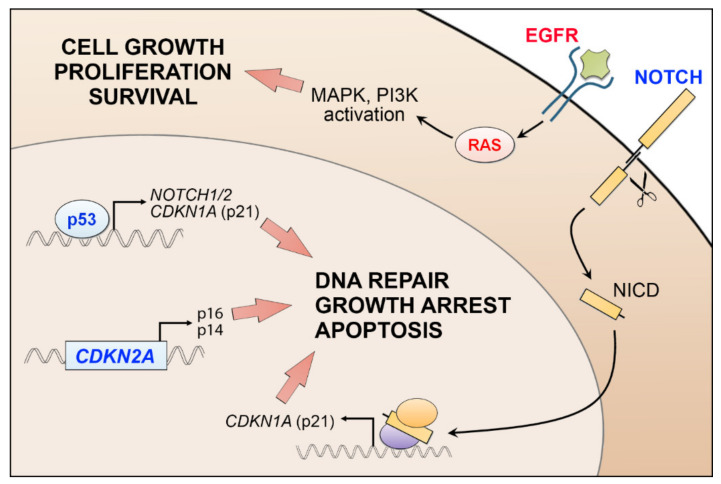
Genetic alterations involved in cSCC progression. Mutational inhibition of wild-type function (in blue) of *TP53*, *CDKN2A*, or *NOTCH1/2* predisposes cells to genomic instability and uncontrolled growth as the expression of their downstream targets, cell cycle regulators p21, p14, and p16 is abrogated. Notch is also transcriptionally regulated by p53. Notch intracellular domain (NICD) is cleaved upon ligand binding and transported to the nucleus to regulate gene transcription in co-operation with other factors. Mutational activation (in red) of EGFR or HRAS induces cell proliferation and survival via MAPK and PI3K signaling.

cSCC is a complex malignant tumor with a high level of molecular heterogeneity [36,37,38]. The average mutational frequency in cSCC, over 50 mutations per mega base pair of DNA, is higher than in any other common tumor types, for instance melanoma, lung, or colorectal cancer [37,39]. A typical mutational signature caused by UVB-irradiation, with prevalent C→T transitions, has been detected in cSCC tumors [7,36,37,38,39,40,41,42]. Mutational inactivation of *TP53* in epidermal keratinocytes is an early event in cSCC development (Figure 2) [36,37,38,39,40]. p53 plays an important role in maintaining genomic stability, and its inactivation results in marked accumulation of UV-induced simple mutations [42]. *TP53* mutations are found already in AKs [43,44,45]. In primary cSCCs, a mutation frequency of *TP53* is 50–60% [37,46], while in metastatic cSCCs, nearly 95% of samples show genetic alterations in the *TP53*, highlighting the role of chronic UV exposure in the progression of cSCC [38,39].

Inactivating mutations of the *CDKN2A*, which encodes important cell cycle regulators p16(INK4a) and p14(ARF), are also common in cSCC [36,37,38,39,40,47,48]. Interestingly, inactivation of *CDKN2A* occurs at later stage in the progression from AK to cSCC, since it is not mutated in the sun-exposed normal skin (Figure 2) [49]. A recent meta-analysis of publicly-available sequencing data shows several novel genes that are frequently mutated in cSCC, e.g., *EP300*, *PBRM1*, *USP28*, and *CHUK* [50]. Tumor suppressors p300 and SWI/SNF, and EP300 and PBRM1 are involved in epigenetic regulation of gene expression of Notch1 and in chromatin remodeling [50]. A high frequency of truncating mutations have been found in *USP28*, which encodes a deubiquitinase that stabilizes key proteins involved in DNA repair [50]. *CHUK* encodes IκB kinase α, which is involved in NFκB signaling [50]. Furthermore, comparison of the mutation status of local and metastatic cSCC revealed that mutations in *TP53*, *CDKN2A*, and *TERT* genes are more common in metastatic than in local cSCCs [51]. On the other hand, mutations in *SPEN*, *MLL3*, *NOTCH2*, *MLL2*, *CREBBP*, *SPTA1*, *NF1*, and *EP300* are detected more often in local cSCCs [51]. 

Additional driver mutations detected in cSCC include activation of *PIK3CA* and *HRAS* and inactivation of *NOTCH1, NOTCH2, TGFBR1,* and *TGFBR2* [38,39,52]. *NOTCH1* and *NOTCH2* are mutated in up to 85% of cSCC, resulting in truncated Notch receptors and abrogated signaling [36,37,38,39,40,48,53]. *NOTCH1* mutations occur early in cSCC progression, and mutations of this gene are common in sun-exposed normal skin [37,54]. It is possible that *NOTCH1* mutations precede p53 mutations in cSCC development [55]. Notch1 has been shown to be an important factor in *HRAS* driven keratinocyte carcinogenesis, since the BRAF inhibitor vemurafenib-induced cSCCs with mutated *HRAS* harbor a higher number of *NOTCH1* mutations than sporadic cSCCs [37,56]. The loss of Notch1 combined with oncogenic Hras expression induced formation of aggressive tumors, whereas oncogenic *HRAS* expression alone resulted in small tumors or no tumors at all in a xenograft tumor model [54]. On the other hand, the treatment of the skin of the mouse strain carrying active Hras with TPA led to the development of multiple papillomas, some of which developed further to cSCC, providing evidence for the oncogenic role of Hras in cSCC [57].

Frequently mutated or amplified receptor tyrosine kinase (RTK) genes in cSCC include *EGFR*, *FGFR3*, *KIT*, and *ERBB4* [38,48]. Sustained activity of EGFR can induce Ras signaling and uncontrolled cell proliferation and survival via MAPK and PI3K signaling [58] (Figure 2). STAT3, an important signaling transducer downstream of EGFR, plays an important role in promoting keratinocyte migration in wound healing [58], but aberrant STAT3 activity contributes to skin carcinogenesis [59,60,61]. STAT3 activating mutations have not been found in cSCC, but its tumorigenic function is strongly dependent on activated EGFR signaling [62]. Expression of constitutively active Stat3 in mouse skin promotes rapid progression of highly vascularized and poorly differentiated cSCC tumors [59]. On the other hand, in a murine chemical carcinogenesis model, Stat3-deficient keratinocytes go to apoptosis after DMBA-treatment and tumor formation is completely prevented in Stat3-deficient mice after DMBA/TPA-treatment [63]. Stat3 is also involved in UVB-induced epidermal hyperproliferation in mice, whereas Stat3-deficient keratinocytes are highly sensitive to apoptosis after UVB exposure [64]. UVB exposure rapidly downregulates and inhibits Stat3 activity in normal keratinocytes, whereas in mouse cSCCs, repeated UVB exposure results in constitutive activation Stat3 [65].

In addition to genomic mutations, epigenetic regulation allows cancer cells to regulate gene transcription by chromatin modifications [66]. Hypermethylation of gene promoter and inactivation of genes encoding for tumor suppressors, e.g., inflammasome adaptor ASC, p14(ARF), p16(INK4a), and E-cadherin has been detected in cSCC [47,65,66,67,68,69]. Examination of methylation profiles of AK and cSCC compared to epidermis demonstrate a similar aberrant methylation pattern with cancer-specific features [70]. Furthermore, hypomethylation is associated with aging and sun exposure of the epidermis [70]. The degree of hypomethylation also correlates with clinical measures of photoaging, indicating the role of UV-induced epigenetic deregulation during skin carcinogenesis [70]. In a recent study, two distinct methylation subclasses of keratin gene clusters were detected in AK and cSCC samples, suggesting two different cell types of origin for the observed AK/cSCC subgroups [71]. These subclasses could be classified into more “epidermal stem cell-like” and “differentiated keratinocyte-like” by comparing the methylation patterns to previously published DNA methylation status of enhancers of the H1 human embryonic stem cell (ESC) line and enhancers of normal human keratinocytes, respectively [71]. These findings support a model where AK and cSCC originate from distinct keratinocyte differentiation stages. There is also evidence that UV-induced oxidative damage of proteins can act as a cancer-promoting agent and inducer of genomic instability and in this way promote progression of cSCC [72].

In summary, cutaneous carcinogenesis is a complex process, which involves several genetic alterations in epidermal keratinocytes. In addition, other molecular alterations, for example in noncoding RNAs and in the microenvironment of premalignant lesions, are required for development of invasive and metastatic cSCC [73,74]. The tumor stroma is not static and it may be influenced by genetic and other patient-derived intrinsic factors [75]. For example, the expression of ECM components is differentially regulated, and the number of different cell types may vary between patients [76]. Cancer-associated fibroblasts (CAFs) have been shown to enhance cSCC invasion by increasing the expression of the γ2 chain of laminin-332 in tumor cells via TGF-β signaling [77]. Collagen VII has been shown to regulate TGF-β signaling and in this way suppress tumor vascularization in cSCCs [78]. In addition, loss of collagen XV and collagen XVIII from the basement membrane has been noted at the early stage of cSCC progression, whereas in an invasive tumor, collagen XVIII is produced by cSCC cells, and collagen XV accumulates in the cSCC stroma [79]. 

The combination and order of the molecular alterations in cSCC initiation and progression is not fully understood, as some of these changes are found already in normal sun-exposed skin without any sign of malignancy. It appears that the number of driver mutations per cell is a major contributor to cancer initiation, as these cells are clonally expanding, and their genomic instability results in increased mutational burden [49]. However, it is evident that alterations in the tumor microenvironment are also required to promote the progression of AK to invasive cSCC. 

## 3. p53-cSCC Molecular Background, Mutations, Progression 

### 3.1. The Battle of the p53 Mutants in the Skin

Exposure of skin cells to ionizing radiation, such as solar UVR, results in DNA damage, which leads to stabilization and activation of the p53 protein [80]. Stabilization of p53 occurs as a result of post-translational modifications, such as phosphorylation and ubiquitination, which serve as inactivating signals for proteases that normally degrade p53 to maintain low p53-levels in the skin [81]. Once stabilized, p53 can transcriptionally regulate downstream genes involved in maintaining genomic stability and controlling cell division [80]. p53 functions as a gatekeeper by inhibiting cell growth and restricting the passage of mutated cell progeny, thereby protecting the host from cancer development. However, p53 is a common target for genetic alterations in many cancers, especially in UV-induced skin cancers and cSCC [31,32,33,34,35,36,37,38,39,43,44,45,46,80].

In cancer initiation, loss of p53 function allows cells to bypass apoptosis, resulting in clonal expansion of the mutated cells, which precedes cancer development. In accordance with this, UV-induced p53 mutations are frequently found in AK and cSCCIS [43,44,46,82,83], which have the potential to develop into cSCC. The same mutations in cSCC and precursor lesions are observed also in normal chronically sun-exposed skin in areas such as the face, where the patches of p53-mutated cells are larger and more frequent [49,84,85,86,87,88]. The normal sun-exposed human epidermis is a patchwork of competing mutant keratinocyte clones, but it has a remarkable ability to tolerate these mutations and maintain homeostasis. Normal sun-exposed skin displays a mutational UV signature, with each sun-exposed cell carrying on average more than 10,000 somatic mutations [49]. The number of clonal mutations is higher in normal sun-exposed skin compared to sun-protected skin, and these mutations are particularly targeted to tumor-suppressors p53 and NOTCH1 [16,49]. Accumulation of persistent p53 mutations has been detected in 14% of all epidermal cells in sun-exposed skin of mid-life individuals [86]. Particularly, hotspot mutations R248W and G245D in p53 are highly frequent in normal sun-exposed skin, and they may be signs of the early phase of skin carcinogenesis [87]. 

It has been shown that p53 mutations arise very early in mouse skin after UVB exposure, and that the growth of the p53-mutant keratinocytes is driven by UVB [49,89]. Interestingly, such mutant progenitor cells can colonize and persist in normal human epidermis without forming tumors [90]. A recent study showed that mouse epidermal cells with a single-allele p53 mutation (*trp53^R245W^*, the murine equivalent of human *TP53^R248W^*) can grow over wild-type cells to colonize normal epidermis, but the epidermis can adapt to the mutant clones and revert the expansion of the mutant cells over time [91]. Short-term exposure to UVB significantly accelerated the expansion of the p53-mutant clones, but continuous exposure to UVB resulted in a decline in this mutant population and displacement by other UV-induced mutant clones with a growth advantage over the initial p53-mutant population [88]. What determines the fate of these mutant clones to proceed into skin tumorigenesis is unclear. It is possible that different p53 mutants provide different survival advantages to keratinocytes [92]. In addition, while chronic UV exposure leads to the formation of skin tumors in mice already after 8 weeks, discontinuation of UV treatment delays tumor formation but does not prevent it [92]. 

Normal sun-exposed skin adjacent to a cSCC harbors a high mutation burden, including the UV-targeted genes *TP53* and *NOTCH1/2* [87]. The overall mutation rate is markedly higher in the normal skin adjacent to cSCC in patients with a high burden of skin cancer (severe UV-damage, multiple prior cSCCs and AKs) compared to patients with low burden (only a single diagnosis of cSCC and few AKs) [87]. Particularly, *TP53* is mutated three times more frequently in the normal skin of patients with a high skin cancer burden compared to the low-burden group, indicating that elevated accumulation of UV-signature mutations is associated with an increased burden of cSCC [87].

p53 shares a high degree of structural and functional homology with two of its ancestral genes, p63 and p73 [93]. They are all expressed as several isoforms, categorized in the N-terminal full-length transactivation (TA) and truncated (∆N) isoforms [93]. In addition, several C-terminally spliced isoforms exist, leading to large complexity in biological function of the p53-family proteins [93,94]. Due to sequence similarity in their DNA-binding domain, p63 and p73 can activate many p53-target genes, but they also serve independent functions, for example in embryonic development when distinct isoforms are specifically expressed in different developmental phases [94]. The role of p73 and its different isoforms in cSCC development is not well known, but it has been shown that heterozygous deletion of p53 and p73 (p53^+/−^;p73^+/−^) drives spontaneous cSCC development in mice, indicating that p73 may play a role in cSCC development [94]. The ΔNp63 isoform plays a major role in mediating epidermal development, and it is much more abundant than TAp63, which is mainly expressed in basal epidermal keratinocytes [94].

p63 and p73 are rarely mutated in cancer, but the balance between their different isoforms can be critical for the cell fate and cancer development [94,95]. For example, TAp63 plays a tumor suppressive role in cSCC [96,97], whereas ΔNp63 is tumorigenic [98,99]. Genomic amplification of the *TP63* gene has been reported in cSCCs, and given the substantially higher expression of the ΔNp63 compared to TAp63 in normal epidermis, the oncogenic function of p63 is primarily dependent on the overexpressed ΔNp63 isoform in the skin [98]. The ΔNp63 isoform regulates several transcriptional programs to exert its oncogenic function in cSCC [98]. Moreover, there is a dominant-negative interplay between the wild-type p63/p73 and mutant p53. In its active conformation, p53 forms a tetramer, which can then bind to the target DNA sequence to activate gene transcription [93]. The structural similarity between p63 and p73 allows them to form heterotetramers, but p53 is unable to interact with them. However, mutations in p53 can alter its conformation so that it can interact with p63 or p73 and inhibit their transcriptional activity and this way promote tumorigenesis [93,95]. In addition to blocking p63/p73 transcriptional programs, mutant p53 can use p63 as a molecular chaperone to enable binding of mutant p53 to a specific set of gene promoters and in this way promote oncogenesis [100]. The co-operation of p63 and mutant p53 drives a gene expression pattern to facilitate cancer cell invasion through the release of a pro-invasive secretome [100]. One such target gene, α1-antitrypsin (*A1AT*), which is upregulated through the mut53–p63 complex, promotes tumor invasion by inducing the expression of several epithelial–mesenchymal transition (EMT) markers [101].

Mutations in the *TP53* gene are early events in skin carcinogenesis, and they predispose skin cells to further genomic instability. In accordance with this, several studies show remarkably high overall mutational burden in human cSCCs, which occur mostly at older age [3,4,5,6,7]. Thus, prevention of carcinogenic exposure to the sun’s UV light is the most effective way of preventing the accumulation of mutations in the skin and the development of skin cancers, including cSCC.

### 3.2. p53 Mutations in cSCC 

**Figure 3:**
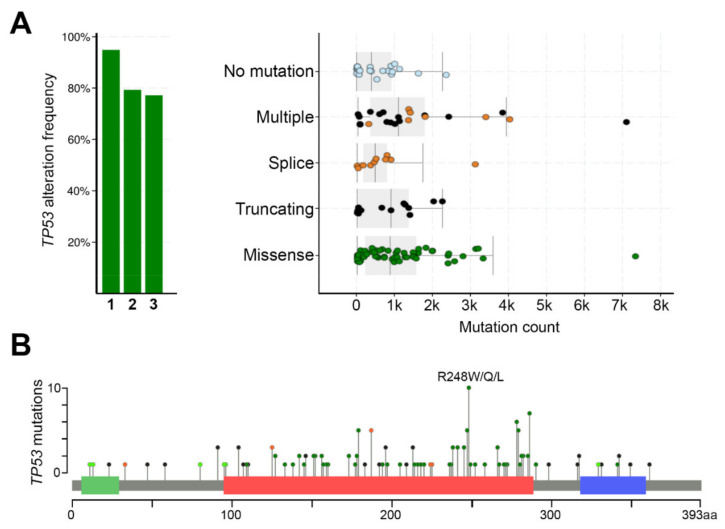
Mutations in *TP53* gene in cSCC in cBioPortal database [102,103]. (**A**) Left: *TP53* alteration frequency in three independent cSCC cohorts comprising a total of 125 samples (1 = MD Anderson [39], 2 = DFCI [38], 3 = UCSF excluding cSCCs with hereditary disorders). Right: Mutation plot showing p53 mutation type and count in cSCCs. (**B**) The number and position of mutations in the *TP53* gene. The green bar indicates p53 transactivating domain, the red bar the DNA-binding domain, and the blue bar the p53 tetramerization motif. The circles are colored with respect to the corresponding mutation types (green = missense, black = truncating, orange = inframe deletion or insertion). The hotspot mutation R248W/Q/L is highlighted in the plot.

**Figure 4:**
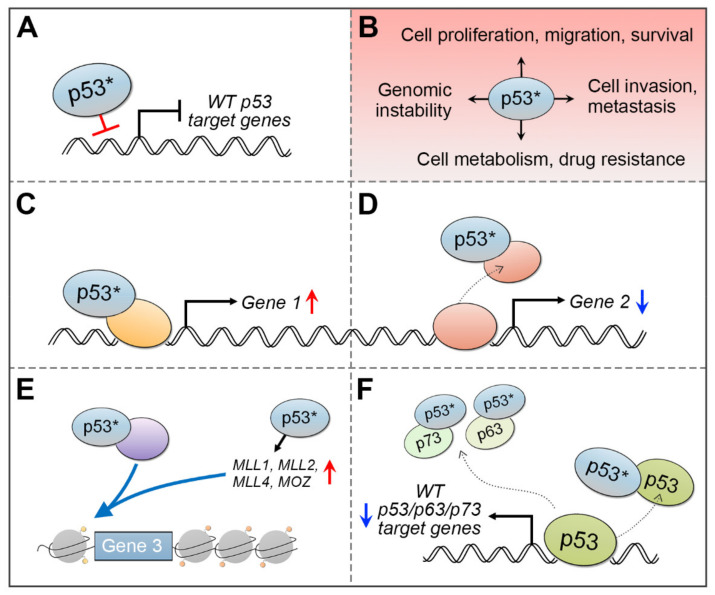
Mutant p53 gain-of-function mechanisms. (**A**) A missense mutation in the TP53 DNA-binding domain (e.g., in amino acid residue 248) can disturb p53 protein binding to gene regulatory areas and prevent transcription of the p53 wild-type target genes. (**B**) Gain-of-function p53 mutants can influence several cellular processes to facilitate cancer progression. (**C**) The mutant p53 (p53*) cannot recognize the p53 DNA response elements, but it can bind to various transcription factors (in orange) and recruit them to gene promoters to activate transcription. (**D**) In some instances, mutant p53 may decoy transcription factors (in red) from their target sites and inhibit transcription. (**E**) Mutant p53 can upregulate expression of chromatin remodeling proteins, such as MLL1, MLL2, MLL4, and MOZ, or bind to and recruit chromatin modifying proteins (in purple, e.g., MLL4 or the SWI/SNF complex) to alter the epigenetic state of the chromatin and make it accessible for gene transcription. (**F**) Mutant p53 may inhibit the wild-type p53, or other p53 family members p63 or p73 (in green) in a dominant-negative manner, where it complexes with the wild-type p53/p63/p73 to block its function and target gene expression.

The mutation rate of *TP53* gene in cSCC tumors is very high, ranging from about 50% in primary cSCCs to nearly 95% in aggressive tumors [36,37,38,39,40,41,42,43,44,45,46,47,48,49]. Skin SCCs have a wide spectrum of point mutations in the *TP53* gene, but not all mutations are equal. As in many other human cancers, also in cSCCs the missense mutations in *TP53* gene are frequently found in the conserved p53 DNA-binding domain (Figure 3). Certain nucleotide positions within this region are mutated with exceptionally high frequency, and such alterations are called hotspot mutations. One such hotspot mutation in cSCC is commonly detected in the amino acid residue 248 (e.g., R248W/R248Q), which makes direct contact with DNA [80]. Thus, the mutated p53 protein fails to bind to DNA and activate its target genes (Figure 4A). This can potentially block p53-mediated arrest of cell growth and apoptosis and lead to accumulation of the mutant p53, partly because it is more stable than the wild-type p53, and with mutant p53, there is no functional negative feedback response by MDM2, the principal antagonist of p53 [30]. 

It has been reported that the mutant p53 can acquire specific gain-of-function (GOF) oncogenic activities, such as increased pro-growth signaling, invasiveness, and tumor metastasis (Figure 4B) [104,105,106]. A distinctive function of the mutant p53 is its ability to associate with other transcription factors, histone-modifying proteins, or the transcription initiation complex to activate transcription [104,105,106]. The GOF activity of the mutant p53 can be exerted by several mechanisms (Figure 4). The mutant p53 can bind to transcription factors to activate (Figure 4C) or inactivate (Figure 4D) target gene expression [104,105,106]. It may increase chromatin accessibility to drive expression of specific genes (Figure 4E) [107,108,109]. Additionally, mutant p53 can disturb the function of wild-type p53 or other p53-family members, p63 or p73, by forming a complex with the mutated p53 and wild-type p53/p63/p73 proteins to inhibit their DNA-binding and target gene activation (Figure 4F) [104,105,106].

The p53^R248W^ contact mutant is commonly detected in cSCCs (Figure 3), but the same mutation is also present in sun-exposed normal skin, AKs, and cSCCIS [44,46,49,87,110]. This p53 mutant exhibits altered DNA-binding and GOF activity in many cancers through increased cell proliferation, chromosomal instability, and drug resistance, and it impairs the wild-type p53 function in a dominant negative manner [109,111,112]. Although short-term UV-exposure increases proliferation of cells harboring either wild-type or mutant p53, the p53^R248W^ mutant progenitor cells have growth advantages over the wild-type cells and are able to colonize normal epidermis [91]. 

Although normal skin is able to tolerate the p53 mutant keratinocytes to a certain extent, the presence of the p53^R248W^ mutant may increase the likelihood of aberrant cell behavior through the GOF activity of the mutant p53 and acquisition of additional mutations. Thus, this particular hotspot mutation could be depicted as a potent and early driver for cSCC development. 

In addition to the contact mutant p53 (e.g., R248W/R248Q), missense mutations in the *TP53* gene, such as R175H, lead to impaired zinc binding and prevent proper p53 protein folding. Similarly to the contact mutant, the conformational p53 mutant loses its affinity to the wild-type p53 DNA response elements. R175H is a *TP53* hotspot mutation in head and neck SCCs (HNSCCs) [113], and it is also detected in cSCCs [37], although it does not qualify as a hotspot mutation in this cancer type. Mutant p53^R175H^ exhibits GOF activity in many cancer types. For example, in HNSCC cells, p53^R175H^ drives the expression of oncogenic transcription factor FOXM1, which is upregulated in human oral premalignant and HNSCC tissues [114]. Mutant p53^R175H^ has been shown to interact with other p53 family members, p63 and p73, and inhibit their transcriptional program to promote cell invasion [95,97,115,116]. In addition, mutant p53^R175H^ drives cSCC development in mice, when specifically expressed in the epidermis [117]. This p53 mutant has been shown to increase skin tumor formation and genomic instability, even when compared to loss of p53 [118,119]. 

In addition to the missense mutations in the *TP53* gene, a significant fraction of human cancers carry *TP53* nonsense mutations, which introduce a premature stop-codon [120]. This will lead to translation termination and p53 transcript degradation via the nonsense-mediated decay (NMD) pathway. These nonsense mutations can vary in different cancer types, e.g., R196* is the most prevalent nonsense mutation in skin cancer, while E298* is the top nonsense mutation in HNSCC [121]. These nonsense p53 or p53 null tumors show negative p53 expression by immunostaining, indicating impaired p53 protein translation due to the mutation [121]. Expectedly, there is a shift towards potent malignant cell transformation due to the absence of p53, as demonstrated in p53-deficient mouse models [119,122,123,124,125,126]. However, emerging evidence suggests that this is not the whole truth. Several isoforms are produced from the TP53 gene, and particularly the truncated isoforms from nonsense-mutated TP53 have been implicated in cancer [127]. Not all truncated isoforms undergo NMD, and some can partially escape degradation. As a result, they can have an independent function distinct from the transcriptional activity of the p53 protein. Indeed, it has been shown that a truncated p53 isoform can specifically localize to mitochondria and bind to cyclophilin D (CypD), leading to increased mitochondria permeability and higher levels of mitochondrial reactive oxygen species [128,129]. Interestingly, this isoform is specifically expressed during tissue injury and in tumors characterized by increased metastasis [128]. In addition, the truncated p53 isoform is capable of reprogramming cells towards mesenchymal-like features [128]. In accordance with this, another study found a similar mechanism for truncated p53 isoforms, which were required for cancer cell survival, and when ectopically expressed in cells could reprogram cells towards pro-metastatic features [129].

In summary, distinct p53 mutants possess a plethora of functional mechanisms in which they can regulate several hallmarks of cancer, from activation of cell proliferation to promotion of invasion and metastasis. The biological consequence of distinct p53 mutants is very context dependent. Moreover, the recent evidence of the cancer-associated p53 isoforms, particularly the truncated ones which can escape degradation, indicates that the loss of function or nonsense p53 mutants should be regarded as an additional subgroup of GOF p53 mutants. Yet, the existence and potential function of different p53 isoforms in the process of skin carcinogenesis is not known.

### 3.3. Mutationally Inactivated p53 Drives cSCC Progression

Mutated *TP53* plays a significant role in cSCC development. Mutant p53 is stable and accumulates in the nucleus, and a strong p53 immunopositivity is a widely used characteristic to detect mutated p53 in tumor tissues [121,130]. Conversely, cells in the normal tissues show weak or absent immunopositivity [81,102]. The proportion of p53 mutations, which corresponds to increased p53 immunopositivity, has been shown to increase from normal-appearing skin to sun-damaged skin and to AK [81]. While p53 immunopositivity and *TP53* mutations are frequently detected in normal sun-exposed skin and AKs, increased p53 immunopositivity in AKs is associated with increasing severity of dysplasia, and with progression of AK to cSCC [83,84,88,89,131]. These results suggest that *TP53* mutations present an early indication of UV damage, and increased *TP53* mutational frequency is associated with potential cSCC development. However, there is also evidence that UV-induced mutation and inactivation of *NOTCH1* may precede mutation of *TP53* [55].

The protective role of tumor suppressor p53 in skin cancer has been shown in several studies. Loss of p53 accelerates skin tumor formation after UV irradiation [123], and loss of p53 in mouse epidermis results in spontaneous skin tumor development even without UV exposure [125,132]. In a mouse chemical skin carcinogenesis model, loss of p53 does not increase the number or growth rate or skin tumors, but it leads to more rapid malignant progression of skin papillomas compared to mice carrying wild-type or heterozygous p53 [122,132]. In addition, GOF mutant p53 has been shown to promote skin tumor formation, and these tumors are poorly differentiated and metastatic [103,124,128,129]. In accordance with this, GOF p53 mutant cSCC tumors show higher levels of cyclin D1 [103] and enriched integrin and Rho signaling as a sign of increased cell proliferation and metastatic potential [118]. Overall, these studies clearly indicate that either loss of p53 or presence of mutant p53 can be regarded as key drivers of cSCC progression. Particularly, the GOF p53 mutant is associated with poor prognosis compared to loss of p53 in skin carcinogenesis [118,119], suggesting GOF mutant p53 proteins as useful therapeutic targets in advanced cSCC.

Human *CDKN2A* gene encodes two important tumor suppressors, p16(INK4a) and p14(ARF), which are frequently inactivated in cSCC (Figure 2) [36,37,38,39,43,44]. As described above, GOF p53 mutant has been shown to drive cSCC formation in mice. Interestingly, high levels of p16(INK4A) were noted in non-metastatic, poorly differentiated SCCs when compared to well differentiated SCCs, suggesting that activation of p16(INK4A) induced by mutant p53 would prevent malignant progression and metastasis of these tumors [103]. Accordingly, co-deletion of the *CDKN2A* gene in the p53 GOF-induced tumors resulted in marked increase in metastasis rate and in a shorter survival in mice when compared with tumors in which Cdkn2a was deleted in the presence of a p53 loss-of-function mutation or wild-type p53 [103]. This study further strengthens the evidence for the function of *CDKN2A* in tumor suppression. In addition, while loss of p53 drives spontaneous cSCC tumor formation, co-deletion of p53 and α_v_ integrin genes in mouse epithelia has been shown to induce development of cSCC [123]. Tumors lacking both p53 and α_v_ integrin in the epithelia showed high Akt activity and decreased immune cell infiltration, but these tumors grew more slowly than tumors which expressed p53 or α_v_ integrin [126]. In this study, elevated α_v_ integrin levels were detected in mice with advanced cSCCs, in correlation with the observation that high levels of α_v_ integrin are detected in the invading margin of the human SCCs, suggesting that reactivation of α_v_ integrin expression in established tumors may facilitate cSCC growth. 

Specific p53 mutants exert different biologic effects. Moreover, the availability of mutant p53 interacting proteins can be context-dependent and vary not only by cell type but also based on the surrounding tumor microenvironment. Indeed, the GOF p53 mutant can mediate several oncogenic functions of the cancer cell itself, and increasing evidence shows that mutant p53 proteins can alter the cancer cell secretome to regulate the tumor microenvironment and promote cancer progression [133]. Matrix metalloproteinases (MMPs) play an important role in tumor invasion by proteolytic remodeling of the ECM, and they can be produced by the tumor cells, surrounding stromal fibroblasts, and by tumor-associated inflammatory cells [134]. The activity of MMPs is regulated by several factors, including specific tissue inhibitors of metalloproteinases (TIMPs) [134]. It has been shown that wild-type p53 potently inhibits MMP-13 and MMP-1 expression by SCC cells, and this results in decreased SCC cell invasion independently of the proapoptotic effect of p53 [135]. In addition, adenoviral delivery of the wild-type p53 in human cSCC xenografts was shown to decrease tumor growth significantly [136]. Interestingly, adenoviral TIMP-3 expression was shown to inhibit SCC tumor growth even more potently than the wild-type p53 alone [136]. This could be due to a more widespread bystander effect of secreted TIMP-3, as compared with the intracellular tumor suppressor p53, but the study clearly demonstrates how tumor growth can be suppressed by manipulating the activity of MMPs in the tumor microenvironment. While wild-type p53 exhibits an anti-invasive role in cSCC, it remains to be elucidated how distinct GOF p53 mutants mediate the tumor secretome and progression of cSCC.

### 3.4. Non-Coding RNAs Take Part in the p53 Signaling Network

When the genetic information is converted from DNA to mRNA, not all mRNAs are translated into proteins. In fact, a significant part of the human genome is transcribed into non-coding RNAs (ncRNAs), which constitute an essential layer of gene regulation [137,138]. MicroRNAs (miRNAs) and long noncoding RNAs (lncRNAs) are two of the most well-known ncRNA subgroups, but several other functionally and structurally distinct subgroups exist, e.g., piwi-interacting RNAs (piRNAs), small nuclear and nucleolar RNAs (snRNAs and snoRNAs), and transfer RNA-derived small RNAs (tsRNAs or tRFs) [137,139]. Non-coding RNAs are important gene regulators in normal cell homeostasis, but they also contribute to pathologic processes in a context-dependent manner, and deregulation of ncRNAs is widely observed in different cancers [140].

MiRNAs are evolutionarily conserved, single-stranded ncRNAs ~22 nucleotides in length. They inhibit gene expression specifically by binding to the 3’-untranslated region of the target mRNA in the cytoplasm, resulting in translational repression or degradation of target mRNA [141]. Compared to miRNAs, lncRNAs are less conserved, much larger in size (>200 nucleotides), and they can be found in any compartment in the cell. They can participate in gene regulation on several levels, from chromatin remodeling, transcriptional, and post-transcriptional gene regulation to protein translation and transport [138]. Recent findings have elucidated the importance of ncRNAs in p53-response, as they can regulate p53 or its downstream targets, and ncRNAs themselves, e.g., miRNA miR-34 and lncRNA *NORAD* (noncoding RNA activated by DNA damage), are direct transcriptional targets of p53 [142,143].

As discussed above, the tumor-suppressive function of p53 is frequently lost in cancer, particularly in skin cancers, and mutated p53 can acquire oncogenic GOF activity. In addition to the proteins that cooperate with mutated p53 to transactivate specific target genes in cancer, also ncRNAs have been shown to participate in the pathogenic signaling via p53 mutants [144]. As an example, lncRNA *MALAT1* (metastasis associated lung adenocarcinoma transcript 1), which is commonly upregulated in cancer, has been shown to interact with mutated p53 in breast cancer [142]. Here, oncogenic splicing factor SRSF1 bridges *MALAT1* to mutant p53-ID4 protein complex, delocalizing *MALAT1* from nuclear speckles and favoring its association with chromatin [145]. This will lead to alternative splicing and expression of pro-angiogenic VEGFA isoforms in breast cancer [145].

While the role of ncRNAs in cSCC progression is emerging [74,146], only few studies have reported the relationship between p53 and ncRNAs in cSCC progression. Recently, miR-216b has been indicated to have a tumor suppressive function in cSCC. Its expression is decreased in cSCC, while its target gene *TPX2* (TPX2 microtubule nucleation factor) is upregulated [147]. High nuclear TPX2 expression has been shown to correlate with *TP53* mutation and aggressive clinical behavior in breast cancer, supporting a TPX2-p53 regulatory circuit [148]. In cSCC cells, the expression of p53 and its downstream target p21 was increased in the presence of miR-216b mimic, while TPX2 expression was decreased. This was accompanied by reduced cSCC cell growth, migration, and invasion, indicating that miR-216b activates p53 signaling by targeting TPX2 in cSCC [148]. Another study shows that microRNAs miR-30c-2* and miR-497 are important players in suppressing cSCC progression and metastasis, and these miRNAs are regulated by TAp63, a member of the p53 family with a potent tumor-suppressive role in cancer [96].

**Figure 5:**
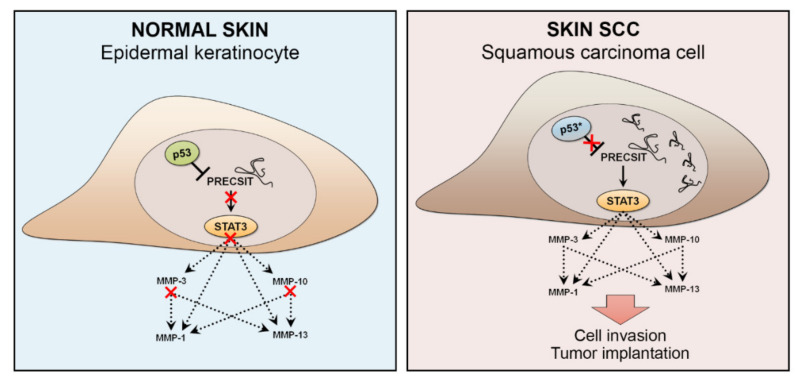
PRECSIT is a p53-regulated lncRNA with a tumorigenic role in cutaneous squamous cell carcinoma (cSCC). Proposed molecular model for the mechanistic role of PRECSIT in cSCC; low level of PRECSIT expression is maintained in normal epidermal keratinocytes by p53. The red cross indicates blockage of the downstream signaling. In cSCC cells, mutational inactivation of p53, which leads to loss of p53 expression, results in upregulation and accumulation of PRECSIT in the nucleus. Elevated PRECSIT expression contributes to STAT3 activation and up-regulation of matrix metalloproteinases collagenase-1 (MMP-1), collagenase-3 (MMP-13), stromelysin-1 (MMP-3), and stromelysin-2 (MMP-10). MMP-3 and MMP-10 are capable of degrading several extracellular matrix (ECM) components, including basement membrane type IV collagen, fibronectin, and laminin. They also activate latent collagenases MMP-1 and MMP-13, capable of cleaving fibrillar collagens type I and III in the dermal ECM. The proteolytic remodeling of ECM and the basement membrane by MMPs is essential for cSCC cell invasion and tumor cell implantation.

MiRNA regulation is very context-dependent, and certain miRNAs are specifically regulated by mutant p53. For example, miR-1246 is induced by mutant p53 in colon cancer, and miR-34 is suppressed by mutant p53 in lung cancer [144]. Thus, it is possible that these two miRNAs are regulated in a similar manner in cSCC, as elevated levels of miR-1246 have been detected in cSCC tissue and serum [149,150], whereas miR-34, a downstream target of p53, is downregulated in cSCC [80]. *PRECSIT* (p53 regulated carcinoma-associated STAT3 activating long intergenic non-protein coding transcript) is a recently characterized lncRNA with a tumorigenic function in cSCC [151] (Figure 5). A nonsense mutation in *TP53* can cause premature p53 translation termination, leading to undetected p53 protein expression. Here, *PRECSIT* was shown to be upregulated in cSCC, and its high expression was specifically associated with loss of p53 in cSCC in vivo [151]. This suggests that loss of p53 may promote *PRECSIT* expression in cSCC progression. In accordance with this, delivery of a wild-type p53 into cSCC cells, which harbor mutated p53 led to downregulation of *PRECSIT* expression, indicating that its expression is suppressed by p53. *PRECSIT* was further shown to activate STAT3-signalling and expression of MMPs and invasion of cSCC cells [151] (Figure 5).

Although cSCCs harbor a massive mutational burden, it is not known how these mutations affect ncRNA functions. Deregulation of a specific ncRNA could be a consequence of mutationally activated or inactivated upstream signaling. However, the majority of the UV-induced mutations fall into the non-coding genome, which can affect, e.g., chromatin structure, transcription factor binding, and gene expression [152]. In addition, these mutations may alter lncRNA expression or the secondary structure, or disturb lncRNA interaction with other regulatory factors [153]. Finally, it is becoming evident that analogous to DNA methylation and histone modifications, ncRNAs are also modified by specific enzymes, so called writers, readers, and erasers, revealing a whole new layer of ncRNA regulation—epitranscriptomics [154]. Emerging evidence suggests that RNA modification pathways are dysregulated in cancer, and cancer cells are often specifically addicted to RNA modifying enzymes to sustain cell proliferation and tumor progression [154]. Epitranscriptomics is a rapidly growing field, and RNA modifying enzymes present an attractive new target for drug discovery. 

## 4. Therapeutic Strategies Targeting Mutant p53 in cSCC 

Due to a high mutation burden and presence of different p53 mutations in cSCC, therapeutic targeting of mutated *TP53* has attracted attention as a potential treatment strategy for cSCC. As discussed above, *TP53* mutations accumulate already in normal sun-exposed skin and in AK and cSCCIS lesions, suggesting that *TP53* targeted therapies could be useful already at an earlier stage of the cSCC progression to prevent progression of precancerous lesions to invasive cSCCs [87]. However, evidence for the role of mutated p53 in invasion of cSCC suggests that mutated p53 may also serve as a potential target in advanced and metastatic cSCC [135].

The treatment of primary cSCC is surgical excision of the tumor with adequate margins. When margins are not sufficient and the curation is not achieved, local recurrences can be detected, and the risk for metastasis increases up to 25–45% [31,34,155,156]. If surgical excision is not achievable, primary radiation therapy is an optional treatment [157]. In addition, if total resection of the cSCC tumor is not achieved, if widespread perineural invasion is noted, or if there are metastases, postoperative adjuvant radiation therapy is indicated [157,158,159]. Treatment options for metastatic cSCC are limited, but results with new immunomodulatory therapies are promising [160,161]. Increased expression of programmed cell death protein-1 (PD-1) and PD-ligand 1 (PD-L1) has been noted in cSCC compared to normal skin [162]. Furthermore, PD-L1 expression in cSCC has been shown to be related to the risk of metastasis and correlates with poor prognosis [163]. Cemiplimab is a PD-1 blocking monoclonal antibody, which functions as immune checkpoint inhibitor. Cemiplimab has been approved by the FDA and EMA as the first line treatment for locally advanced and metastatic cSCC, if curative excision or curative radiation therapy is not achieved, representing the only approved systemic therapy for cSCC [157]. It is expected that other checkpoint inhibitors and immunotherapies will follow cemiplimab in the treatment of advanced cSCC [164]. Second-line systemic treatments for patients with advanced cSCCs include EGFR inhibitor (cetuximab) combined with chemotherapy or radiation therapy [157]. Platinum-based chemotherapeutic cisplatin, fluorouracil, and EGFR-inhibitor are utilized in different combinations, although their use is limited due to adverse events and incomplete remission, and especially the use of cisplatin is limited to a small number of patients [157].

The cell cycle arrest and induction of apoptosis by immune checkpoint inhibitors and fluorouracil is p53-dependent, providing a possible explanation to lack of response to these treatments in some patients. Analysis of the *TP53* mutation status could therefore allow a selection of patients to respond to the treatment. Furthermore, adding p53 mutation-targeted treatment could improve the response of non-responder patients to cemiplimab or fluorouracil [165,166,167,168,169].

Several therapeutic compounds targeted to mutated p53 are in preclinical and clinical studies with different cancers [170,171]. The results of preclinical studies have been promising and are therefore interesting with respect to treatment of cSCC [172,173]. One challenge in drug development for cSCC is to target distinct mutants of p53. However, the presence of several missense mutations in p53 in cSCCs makes this approach challenging and emphasizes the importance of patient selection based on p53 mutation status. The strategies for p53 targeted therapy are restoring and reactivation of wt-p53, eliminating GOF activities of mutant p53, or targeting mutant p53-regulated pathways [110,113,174].

Small molecule inhibitors of mutated p53 developed to reactivate wt-p53 are being tested in several clinical trials in different cancers [172,173,174]. One of these, a small molecular weight cysteine-binding compound, CP-31398, targets mutant p53 and makes it refold as wt-p53 and retain the tumor suppressor function. CP-31398 has been shown to block UVB-induced skin carcinogenesis associated with increased p53 and p21 expression and downregulation of cyclin D1 expression. In addition, CP-31398 promoted apoptosis of keratinocytes in UVB-irradiated wt-p53 SKH-1 mice carrying wild-type p53 and in human epidermoid carcinoma cells in vivo, indicating potential blockade of the skin carcinogenesis. However, its precise efficacy in vivo is unclear [175]. Another small molecule, APR-246 targeting mutated p53 has been studied in clinical trials in esophageal adenocarcinoma or SCC in combination with neoadjuvant chemotherapies fluorouracil and cisplatin, which are used also in treatment of non-curable cSCC tumors [174,175,176].

Another therapeutic option is targeting the allele-specific mutants of p53, which are detected commonly in cSCCs, to eliminate GOF activities. Small molecule zinc metallochaperone-1 (ZMC-1) has been shown to restore the proper folding and transcriptional activity of p53 mutant inducing apoptosis in the p53^R175H^ xenograft model [177]. In addition, P53R3, another small molecule, has been shown to restore the DNA-binding ability of p53 mutants p53^R175H^, p53^R248W^, and p53^R273H^ in the p53 null glioma cell line [178]. However, the high mutation rate of TP53 in cSCC represents a challenge for small molecule-based p53 targeted therapies. The therapeutic option for cSCC and AK could be targeting of downstream pathways that are altered by distinct mutants of p53. In addition, p53^R248W^ or p53^R175H^ mutants could be targeted and used topically for treatment of AK or cSCCIS [80,113].

Delivery of the p53 gene directly into tumor cells is one option to target p53 mutations, and wt-p53 can be transferred to cells by viral vector in combination with cancer treatments. Combining chemotherapy, immunotherapy, or radiation therapy with the p53 gene therapy may increase the efficacy of tumor cell targeted therapies. For example, in HNSCCs, intra-tumoral p53 gene delivery is being studied. In phase 2 multi-center open label clinical trial adenoviral p53 (Ad-p53) gene therapy is administered intratumorally in combination with immune checkpoint PD-1 and PD-L1 inhibitors in patients with recurrent or metastatic HNSCC and other solid tumors approved for anti-PD-1 or anti-PD-L1 therapy [179]. Another clinical phase 1/2 study studied the combination of intratumoral injections of Ad-p53 and anti-PD-1 or anti-PD-L1 therapy nivolumab in recurrent HNSCCs. [180]. In addition, the efficacy of p53 gene therapy is being studied in a phase 2 trial for advanced, resectable SCC of the oral cavity, oropharynx, larynx, and pharynx, combining surgical resection and neoadjuvant chemoradiotherapy followed with Ad5CMV-p53 injection [181]. Furthermore, two individual p53 vaccines are currently in phase 1 clinical trials in patients with HNSCC. First, autologous vaccines are designed and patient leucopheresized dendritic cells (DC) are pulsed with wt-p53 peptides with or without T-helper (Th) peptides. Vaccine is administered via intrabdominal injection [182]. Second, combined modified vaccinia virus Ankara vaccine expressing p53 (p53MVA vaccine), and immune checkpoint PD-1 and PD-L1 inhibitor pembrolizumab are being investigated in combination in HNSCC and other incurable solid tumors that have failed prior therapy [183].

In summary, p53 targeting is an interesting area in drug development, but the efficacy of p53 targeted therapies has not yet been tested in advanced and metastatic cSCCs. It is conceivable that drugs targeting several mutants of p53 are needed, and that p53-targeting drugs would be used in combination with surgical excision, immunomodulators, radiation therapy, or chemotherapies. However, given the important role of p53 mutations in the progression of cSCC, it is expected that p53-targeted therapies could be useful already at an earlier stage of cSCC development, in addition to treatment of advanced and metastatic cSCC.

## 5. Conclusions

Cutaneous squamous cell carcinoma (cSCC) is the most common metastatic skin cancer, and it is associated with poor prognosis in the advanced stage. The mutation rate of cSCC is one of the highest among the malignant tumors, and the majority of mutations found in cSCC are UV-induced. An important early event in cSCC development is mutation and inhibition of the wild-type function of tumor suppressor p53. This leads to the accumulation of additional oncogenic mutations. It has become evident that additional alterations, for example in non-coding RNAs, are required for the progression of premalignant lesion and actinic keratosis to invasive and metastatic cSCC. In addition, the role of p53 in the invasion of cSCC has also been elucidated. Together, these observations suggest mutant p53 as a putative target in both at the early stage of cSCC progression, as well as in advanced and metastatic stages. It is possible that multiple p53 targeted therapeutic approaches under development may be feasible in the treatment of cSCC at different stages of tumor progression. 

## Data Availability

No new data were created or analyzed in this study. Data sharing is not applicable to this article.

## References

[1] Nehal K.S., Bichakjian C.K. (2018). Update on keratinocyte carcinomas. N. Engl. J. Med..

[2] Venables Z.C., Autier P., Nijsten T., Wong K.F., Langan S.M., Rous B., Broggio J., Harwood C., Henson K., Proby C.M. (2019). Nationwide incidence of metastatic cutaneous squamous cell carcinoma in England. JAMA Derm..

[3] Venables Z.C., Nijsten T., Wong K.F., Autier P., Broggio J., Deas A., Harwood C.A., Hollestein L.M., Langan S.M., Morgan E. (2019). Epidemiology of basal and cutaneous squamous cell carcinoma in the U.K. 2013–15: A cohort study. Br. J. Dermatol..

[4] Rogers H.W., Weinstock M.A., Feldman S.R., Coldiron B.M. (2015). Incidence Estimate of Nonmelanoma SkinCancer (Keratinocyte Carcinomas) in the U.S. Population, 2012. JAMA Derm..

[5] Green A.C., Olsen C.M. (2017). Cutaneous squamous cell carcinoma: An epidemiological review. Br. J. Derm..

[6] Muzic J.G., Schmitt A.R., Wright A.C., Alniemi D.T., Zubair A.S., Olazagasti Lourido J.M., Sosa Seda I.M., Weaver A.L., Baum C.L. (2017). Incidence and trends of basal cell carcinoma and cutaneous squamous cell carcinoma: A population-based study in Olmsted County, Minnesota, 2000 to 2010. Mayo Clin. Proc..

[7] Knuutila J.S., Riihilä P., Kurki S., Nissinen L., Kähäri V.M. (2020). Risk factors and prognosis for metastatic cutaneous squamous cell carcinoma: A cohort study. Acta Derm. Venereol..

[8] Burton K.A., Ashack K.A., Khachemoune A. (2016). Cutaneous squamous cell carcinoma: A review of high-risk and metastatic disease. Am. J. Clin. Dermatol..

[9] Gurney B., Newlands C. (2014). Management of regional metastatic disease in head and neck cutaneous malignancy. 1. Cutaneous squamous cell carcinoma. Br. J. Oral Maxillofac. Surg..

[10] Wehner M.R., Cidre Serrano W., Nosrati A., Schoen P.M., Chren M.M., Boscardin J., Linos E. (2018). All-cause mortality in patients with basal and squamous cell carcinoma: A systematic review and meta-analysis. J. Am. Acad. Derm..

[11] Manyam B.V., Garsa A.A., Chin R.I., Reddy C.A., Gastman B., Thorstad W., Yom S.S., Nussenbaum B., Wang S.J., Vidimos A.T. (2017). A multi-institutional comparison of outcomes of immunosuppressed and immunocompetent patients treated with surgery and radiation therapy for cutaneous squamous cell carcinoma of the head and neck. Cancer.

[12] Karia P.S., Han J., Schmults C.D. (2013). Cutaneous squamous cell carcinoma: Estimated incidence of disease, nodal metastasis, and deaths from disease in the United States, 2012. J. Am. Acad. Derm..

[13] Schmults C.D., Karia P.S., Carter J.B., Han J., Qureshi A.A. (2013). Factors predictive of recurrence and death from cutaneous squamous cell carcinoma: A 10-year, single-institution cohort study. JAMA Derm..

[14] Nelson T.G., Ashton R.E. (2017). Low incidence of metastasis and recurrence from cutaneous squamous cell carcinoma found in a UK population: Do we need to adjust our thinking on this rare but potentially fatal event?. J. Surg. Oncol..

[15] Kang S.Y., Toland A.E. (2016). High risk cutaneous squamous cell carcinoma of the head and neck. World J. Otorhinolaryngol. Head Neck Surg..

[16] Liang S.B., Ohtsuki Y., Furihata M., Takeuchi T., Iwata J., Chen B.K., Sonobe H. (1999). Sun-exposure- and aging-dependent p53 protein accumulation results in growth advantage for tumour cells in carcinogenesis of nonmelanocytic skin cancer. Virchows Arch..

[17] Ramos J., Villa J., Ruiz A., Armstrong R., Matta J. (2004). UV dose determines key characteristics of nonmelanoma skin cancer. Cancer Epidemiol. Biomark. Prev..

[18] Xiang F., Lucas R., Hales S., Neale R. (2014). Incidence of nonmelanoma skin cancer in relation to ambient UV radiation in white populations, 1978–2012: Empirical relationships. JAMA Derm..

[19] Lindelöf B., Sigurgeirsson B., Gäbel H., Stern R.S. (2000). Incidence of skin cancer in 5356 patients following organ transplantation. Br. J. Derm..

[20] Kivisaari A., Kähäri V.M. (2013). Squamous cell carcinoma of the skin: Emerging need for novel biomarkers. World J. Clin. Oncol..

[21] Harwood A., Mesher D., McGregor J.M., Mitchell L., Leedham-Green M., Raftery M., Cerio R., Leigh I.M., Sasieni P., Proby C.M. (2013). A surveillance model for skin cancer in organ transplant recipients: A 22-year prospective study in an ethnically diverse population. Am. J. Transplant..

[22] Alam M., Ratner D. (2001). Cutaneous squamous-cell carcinoma. N. Engl. J. Med..

[23] Velez N.F., Karia P.S., Vartanov A.R., Davids M.S., Brown J.R., Schmults C.D. (2014). Association of advanced leukemic stage and skin cancer tumor stage with poor skin cancer outcomes in patients with chronic lymphocytic leukemia. JAMA Derm..

[24] Brewer J.D., Shanafelt T.D., Khezri F., Sosa Seda I.M., Zubair A.S., Baum C.L., Arpey C.J., Cerhan J.R., Call T.G., Roenigk R.K. (2015). Increased incidence and recurrence rates of nonmelanoma skin cancer in patients with non-Hodgkin lymphoma: A Rochester Epidemiology Project population-based study in Minnesota. J. Am. Acad. Derm..

[25] Purdie K.J., Proby C.M., Rizvi H., Grin H., Doorbar J., Sommerlad M., Feltkamp M.C., der Meijden E.V., Inman G.J., South A.P. (2018). The role of human papillomaviruses and polyomaviruses in BRAF-Inhibitor induced cutaneous squamous cell carcinoma and benign squamoproliferative lesions. Front. Microbiol..

[26] Arafa A., Mostafa A., Navarini A.A., Dong J.Y. (2020). The association between smoking and risk of skin cancer: A meta-analysis of cohort studies. Cancer Causes Control..

[27] Parekh V., Seykora J.T. (2017). Cutaneous squamous cell carcinoma. Clin. Lab. Med..

[28] Madani S., Marwaha S., Dusendang J.R., Alexeeff S., Pham N., Chen E.H., Han S., Herrinton L.J. (2021). Ten-year follow-up of persons with sun-damaged skin associated with subsequent development of cutaneous squamous cell carcinoma. JAMA Derm..

[29] Levine D.E., Karia P.S., Schmults C.D. (2015). Outcomes of patients with multiple cutaneous squamous cell carcinomas: A 10-year single-institution cohort study. JAMA Derm..

[30] Thompson A.K., Kelley B.F., Prokop L.J., Murad M.H., Baum C.L. (2016). Risk factors for cutaneous squamous cell carcinoma recurrence, metastasis, and disease-specific death: A systematic review and meta-analysis. JAMA Derm..

[31] Baum C.L., Wright A.C., Martinez J.C., Arpey C.J., Brewer J.D., Roenigk R.K., Otley C.C. (2018). A new evidence-based risk stratification system for cutaneous squamous cell carcinoma into low, intermediate, and high risk groups with implications for management. J. Am. Acad. Derm..

[32] Que S.K.T., Zwald F.O., Schmults C.D. (2018). Cutaneous squamous cell carcinoma: Incidence, risk factors, diagnosis, and staging. J Am. Acad. Dermatol..

[33] Roscher I., Falk R.S., Vos L., Clausen O.P.F., Helsing P., Gjersvik P., Robsahm T.E. (2018). Validating 4 staging systems for cutaneous squamous cell carcinoma using population-based data: A nested case-control study. JAMA Derm..

[34] Zeng S., Fu L., Zhou P., Ling H. (2020). Identifying risk factors for the prognosis of head and neck cutaneous squamous cell carcinoma: A systematic review and meta-analysis. PLoS ONE.

[35] Motley R., Kersey P., Lawrence C. (2002). Multiprofessional guidelines for the management of the patient with primary cutaneous squamous cell carcinoma. Br. J. Derm..

[36] Inman G.J., Wang J., Nagano A., Alexandrov L.B., Purdie K.J., Taylor R.G., Sherwood V., Thomson J., Hogan S., Spender L.C. (2018). The genomic landscape of cutaneous SCC reveals drivers and a novel azathioprine associated mutational signature. Nat. Commun..

[37] South A.P., Purdie K.J., Watt S.A., Haldenby S., den Breems N., Dimon M., Arron S.T., Kluk M.J., Aster J.C., McHugh A. (2014). NOTCH1 mutations occur early during cutaneous squamous cell carcinogenesis. J. Invest. Dermatol..

[38] Li Y.Y., Hanna G.J., Laga A.C., Haddad R.I., Lorch J.H., Hammerman P.S. (2015). Genomic analysis of metastatic cutaneous squamous cell carcinoma. Clin. Cancer. Res..

[39] Pickering C.R., Zhou J.H., Lee J.J., Drummond J.A., Peng S.A., Saade R.E., Tsai K.Y., Curry J.L., Tetzlaff M.T., Lai S.Y. (2014). Mutational landscape of aggressive cutaneous squamous cell carcinoma. Clin. Cancer Res..

[40] Cho R.J., Alexandrov L.B., den Breems N.Y., Atanasova V.S., Farshchian M., Purdom E., Nguyen T.N., Coarfa C., Rajapakshe K., Prisco M. (2018). APOBEC mutation drives early-onset squamous cell carcinomas in recessive dystrophic epidermolysis bullosa. Sci. Transl. Med..

[41] Mueller S.A., Gauthier M.A., Ashford B., Gupta R., Gayevskiy V., Ch’ng S., Palme C.E., Shannon K., Clark J.R., Ranson M. (2019). Mutational patterns in metastatic cutaneous squamous cell carcinoma. J. Invest. Dermatol..

[42] Durinck S., Ho C., Wang N.J., Liao W., Jakkula L.R., Collisson E.A., Pons J., Chan S.W., Lam E.T., Chu C. (2011). Temporal dissection of tumorigenesis in primary cancers. Cancer Discov..

[43] Campbell C., Quinn A.G., Ro Y.S., Angus B., Rees J.L. (1993). p53 mutations are common and early events that precede tumor invasion in squamous cell neoplasia of the skin. J. Invest. Dermatol..

[44] Ziegler A., Jonason A.S., Leffell D.J., Simon J.A., Sharma H.W., Kimmelman J., Remington L., Jacks T., Brash D.E. (1994). Sunburn and p53 in the onset of skin cancer. Nature.

[45] Taguchi M., Watanabe S., Yashima K., Murakami Y., Sekiya T., Ikeda S. (1994). Aberrations of the Tumor Suppressor P53 Gene and P53 Protein in Solar Keratosis in Human Skin. J. Invest. Dermatol..

[46] Yilmaz A.S., Ozer H.G., Gillespie J.L., Allain D.C., Bernhardt M.N., Furlan K.C., Castro L.T., Peters S.B., Nagarajan P., Kang S.Y. (2017). Differential mutation frequencies in metastatic cutaneous squamous cell carcinomas versus primary tumors. Cancer.

[47] Brown V.L., Harwood C.A., Crook T., Cronin J.G., Kelsell D.P., Proby C.M. (2004). p16INK4a and p14ARF tumor suppressor genes are commonly inactivated in cutaneous squamous cell carcinoma. J. Invest. Dermatol..

[48] Al-Rohil R.N., Tarasen A.J., Carlson J.A., Wang K., Johnson A., Yelensky R., Lipson D., Elvin J.A., Vergilio J.A., Ali S.M. (2016). Evaluation of 122 advanced-stage cutaneous squamous cell carcinomas by comprehensive genomic profiling opens the door for new routes to targeted therapies. Cancer.

[49] Martincorena I., Roshan A., Gerstung M., Ellis P., Van Loo P., McLaren S., Wedge D.C., Fullam A., Alexandrov L.B., Tubio J.M. (2015). High burden and pervasive positive selection of somatic mutations in normal human skin. Science.

[50] Chang D., Shain A.H. (2021). The landscape of driver mutations in cutaneous squamous cell carcinoma. NPJ Genom. Med..

[51] Lobl M.B., Clarey D., Schmidt C., Wichman C., Wysong A. (2021). Analysis of mutations in cutaneous squamous cell carcinoma reveals novel genes and mutations associated with patient-specific characteristics and metastasis: A systematic review. Arch. Dermatol. Res..

[52] Cammareri P., Rose A.M., Vincent D.F., Wang J., Nagano A., Libertini S., Ridgway R.A., Athineos D., Coates P.J., McHugh A. (2016). Inactivation of TGFβ receptors in stem cells drives cutaneous squamous cell carcinoma. Nat. Commun..

[53] Wang N.J., Sanborn Z., Arnett K.L., Bayston L.J., Liao W., Proby C.M., Leigh I.M., Collisson E.A., Gordon P.B., Jakkula L. (2011). Loss-of-function mutations in Notch receptors in cutaneous and lung squamous cell carcinoma. Proc. Natl. Acad. Sci. USA.

[54] Lefort K., Mandinova A., Ostano P., Kolev V., Calpini V., Kolfschoten I., Devgan V., Lieb J., Raffoul W., Hohl D. (2007). Notch1 is a p53 target gene involved in human keratinocyte tumor suppression through negative regulation of ROCK1/2 and MRCKalpha kinases. Genes Dev..

[55] Su F., Viros A., Milagre C., Trunzer K., Bollag G., Spleiss O., Reis-Filho J.S., Kong X., Koya R.C., Flaherty K.T. (2012). RAS mutations in cutaneous squamous-cell carcinomas in patients treated with BRAF inhibitors. N. Engl. J. Med..

[56] Zheng Q., Capell B.C., Parekh V., O’Day C., Atillasoy C., Bashir H.M., Yeh C., Shim E.H., Prouty S.M., Dentchev T. (2021). Whole-exome and transcriptome analysis of uv-exposed epidermis and carcinoma in situ reveals early drivers of carcinogenesis. J. Invest. Dermatol..

[57] Leder A., Kuo A., Cardiff R.D., Sinn E., Leder P. (1990). v-Ha-ras transgene abrogates the initiation step in mouse skin tumorigenesis: Effects of phorbol esters and retinoic acid. Proc. Natl. Acad. Sci. USA..

[58] Doma E., Rupp C., Baccarini M. (2013). EGFR-ras-raf signaling in epidermal stem cells: Roles in hair follicle development, regeneration, tissue remodeling and epidermal cancers. Int. J. Mol. Sci..

[59] Sano S., Itami S., Takeda K., Tarutani M., Yamaguchi Y., Miura H., Yoshikawa K., Akira S., Takeda J. (1999). Keratinocyte-specific ablation of Stat3 exhibits impaired skin remodeling, but does not affect skin morphogenesis. EMBO J..

[60] Chan K.S., Sano S., Kataoka K., Abel E., Carbajal S., Beltran L., Clifford J., Peavey M., Shen J., Digiovanni J. (2008). Forced expression of a constitutively active form of Stat3 in mouse epidermis enhances malignant progression of skin tumors induced by two-stage carcinogenesis. Oncogene.

[61] Kataoka K., Kim D.J., Carbajal S., Clifford J.L., DiGiovanni J. (2008). Stage-specific disruption of Stat3 demonstrates a direct requirement during both the initiation and promotion stages of mouse skin tumorigenesis. Carcinogenesis.

[62] Sano S., Chan K.S., DiGiovanni J. (2008). Impact of Stat3 activation upon skin biology: A dichotomy of its role between homeostasis and diseases. J. Dermatol. Sci..

[63] Chan K.S., Sano S., Kiguchi K., Anders J., Komazawa N., Takeda J., DiGiovanni J. (2004). Disruption of Stat3 reveals a critical role in both the initiation and the promotion stages of epithelial carcinogenesis. J. Clin. Invest..

[64] Kim D.J., Angel J.M., Sano S., DiGiovanni J. (2009). Constitutive activation and targeted disruption of signal transducer and activator of transcription 3 (Stat3) in mouse epidermis reveal its critical role in UVB-induced skin carcinogenesis. Oncogene.

[65] Sano S., Chan K.S., Kira M., Kataoka K., Takagi S., Tarutani M., Itami S., Kiguchi K., Yokoi M., Sugasawa K. (2005). Signal transducer and activator of transcription 3 is a key regulator of keratinocyte survival and proliferation following UV irradiation. Cancer Res..

[66] Dawson M.A. (2017). The cancer epigenome: Concepts, challenges, and therapeutic opportunities. Science.

[67] Murao K., Kubo Y., Ohtani N., Hara E., Arase S. (2006). Epigenetic abnormalities in cutaneous squamous cell carcinomas: Frequent inactivation of the RB1/p16 and p53 pathways. Br. J. Dermatol..

[68] Meier K., Drexler S.K., Eberle F.C., Lefort K., Yazdi A.S. (2016). Silencing of ASC in cutaneous squamous cell carcinoma. PLoS ONE.

[69] Chiles M.C., Ai L., Zuo C., Fan C.Y., Smoller B.R. (2003). E-cadherin promoter hypermethylation in preneoplastic and neoplastic skin lesions. Mod. Pathol..

[70] Vandiver A.R., Irizarry R.A., Hansen K.D., Garza L.A., Runarsson A., Li X., Chien A.L., Wang T.S., Leung S.G., Kang S. (2015). Age and sun exposure-related widespread genomic blocks of hypomethylation in nonmalignant skin. Genome Biol..

[71] Rodríguez-Paredes M., Bormann F., Raddatz G., Gutekunst J., Lucena-Porcel C., Köhler F., Wurzer E., Schmidt K., Gallinat S., Wenck H. (2018). Methylation profiling identifies two subclasses of squamous cell carcinoma related to distinct cells of origin. Nat. Commun..

[72] Tramutola A., Falcucci S., Brocco U., Triani F., Lanzillotta C., Donati M., Panetta C., Luzi F., Iavarone F., Vincenzoni F. (2020). Protein oxidative damage in uv-related skin cancer and dysplastic lesions contributes to neoplastic promotion and progression. Cancers.

[73] Nissinen L., Farshchian M., Riihilä P., Kähäri V.M. (2016). New perspectives on role of tumor microenvironment in progression of cutaneous squamous cell carcinoma. Cell Tissue Res..

[74] Piipponen M., Nissinen L., Kähäri V.M. (2020). Long non-coding RNAs in cutaneous biology and keratinocyte carcinomas. Cell Mol. Life Sci..

[75] Ji A.L., Rubin A.J., Thrane K., Jiang S., Reynolds D.L., Meyers R.M., Guo M.G., George B.M., Mollbrink A., Bergenstråhle J. (2020). Multimodal Analysis of Composition and Spatial Architecture in Human Squamous Cell Carcinoma. Cell.

[76] Amôr N.G., Santos P.S.D.S., Campanelli A.P. (2021). The tumor microenvironment in scc: Mechanisms and therapeutic opportunities. Front. Cell. Dev. Biol..

[77] Siljamäki E., Rappu P., Riihilä P., Nissinen L., Kähäri V.M., Heino J. (2020). H-Ras activation and fibroblast-induced TGF-β signaling promote laminin-332 accumulation and invasion in cutaneous squamous cell carcinoma. Matrix Biol..

[78] Martins V.L., Caley M.P., Moore K., Szentpetery Z., Marsh S.T., Murrell D.F., Kim M.H., Avari M., McGrath J.A., Cerio R. (2015). Suppression of tgfβ and angiogenesis by type vii collagen in cutaneous scc. J. Natl. Cancer Inst..

[79] Karppinen S.M., Honkanen H.K., Heljasvaara R., Riihilä P., Autio-Harmainen H., Sormunen R., Harjunen V., Väisänen M.R., Väisänen T., Hurskainen T. (2016). Collagens XV and XVIII show different expression and localisation in cutaneous squamous cell carcinoma: Type XV appears in tumor stroma, while XVIII becomes upregulated in tumor cells and lost from microvessels. Exp. Dermatol..

[80] Levine A.J. (1997). P53, the Cellular gatekeeper for growth and division. Cell.

[81] Liu Y., Tavana O., Gu W. (2019). P53 modifications: Exquisite decorations of the powerful guardian. J. Mol. Cell Biol..

[82] Einspahr J.G., Alberts D.S., Warneke J.A., Bozzo P., Basye J., Grogan T.M., Nelson M.A., Bowden G.T. (1999). Relationship of P53 mutations to epidermal cell proliferation and apoptosis in human uv-induced skin carcinogenesis. Neoplasia.

[83] Javor S., Gasparini G., Biatta C.M., Cozzani E., Cabiddu F., Ravetti J.L., Vellone V.G., Parodi A. (2021). P53 staining index and zonal staining patterns in actinic keratoses. Arch. Dermatol. Res..

[84] Albibas A.A., Rose-Zerilli M.J.J., Lai C., Pengelly R.J., Lockett G.A., Theaker J., Ennis S., Holloway J.W., Healy E. (2018). Subclonal evolution of cancer-related gene mutations in p53 immunopositive patches in human skin. J. Invest. Dermatol..

[85] Yizhak K., Aguet F., Kim J., Hess J.M., Kübler K., Grimsby J., Frazer R., Zhang H., Haradhvala N.J., Rosebrock D. (2019). RNA sequence analysis reveals macroscopic somatic clonal expansion across normal tissues. Science.

[86] Fowler J.C., King C., Bryant C., Hall M.W.J., Sood R., Ong S.H., Earp E., Fernandez-Antoran D., Koeppel J., Dentro S.C. (2021). Selection of oncogenic mutant clones in normal human skin varies with body site. Cancer Discov..

[87] Wei L., Christensen S.R., Fitzgerald M.E., Graham J., Hutson N.D., Zhang C., Huang Z., Hu Q., Zhan F., Xie J. (2021). Ultradeep sequencing differentiates patterns of skin clonal mutations associated with sun-exposure status and skin cancer burden. Sci. Adv..

[88] Jonason A.S., Kunala S., Price G.J., Restifo R.J., Spinelli H.M., Persing J.A., Leffell D.J., Tarone R.E., Brash D.E. (1996). Frequent clones of p53-mutated keratinocytes in normal human skin. Proc. Natl. Acad. Sci. USA.

[89] Ståhl P.L., Stranneheim H., Asplund A., Berglund L., Pontén F., Lundeberg J. (2011). Sun-Induced nonsynonymous p53 mutations are extensively accumulated and tolerated in normal appearing human skin. J. Invest. Dermatol..

[90] Kramata P., Lu Y.-P., Lou Y.-R., Singh R.N., Kwon S.M., Conney A.H. (2005). Patches of Mutant P53-immunoreactive epidermal cells induced by chronic uvb irradiation harbor the same p53 mutations as squamous cell carcinomas in the skin of hairless SKH-1 mice. Cancer Res..

[91] Murai K., Skrupskelyte G., Piedrafita G., Hall M., Kostiou V., Ong S.H., Nagy T., Cagan A., Goulding D., Klein A.M. (2018). Epidermal tissue adapts to restrain progenitors carrying clonal p53 mutations. Cell Stem Cell.

[92] Melnikova V.O., Pacifico A., Chimenti S., Peris K., Ananthaswamy H.N. (2005). Fate of UVB-Induced P53 mutations in SKH-Hr1 mouse skin after discontinuation of irradiation: Relationship to skin cancer development. Oncogene.

[93] Chillemi G., Kehrloesser S., Bernassola F., Desideri A., Dötsch V., Levine A.J., Melino G. (2017). Structural evolution and dynamics of the P53 proteins. Cold Spring Harb. Perspect. Med..

[94] Botchkarev V.A., Flores E.R. (2014). P53/P63/P73 in the epidermis in health and disease. Cold Spring Harb. Perspect. Med..

[95] Billant O., Léon A., Le Guellec S., Friocourt G., Blondel M., Voisset C. (2016). The Dominant-negative interplay between P53, P63 and P73: A family affair. Oncotarget.

[96] Davis A.J., Tsinkevich M., Rodencal J., Abbas H.A., Su X.-H., Gi Y.-J., Fang B., Rajapakshe K., Coarfa C., Gunaratne P.H. (2020). TAp63-Regulated mirnas suppress cutaneous squamous cell carcinoma through inhibition of a network of cell-cycle genes. Cancer Res..

[97] Muller P.A.J., Caswell P.T., Doyle B., Iwanicki M.P., Tan E.H., Karim S., Lukashchuk N., Gillespie D.A., Ludwig R.L., Gosselin P. (2009). Mutant P53 drives invasion by promoting integrin recycling. Cell.

[98] Gatti V., Fierro C., Annicchiarico-Petruzzelli M., Melino G., Peschiaroli A. (2019). ΔNp63 in squamous cell carcinoma: Defining the oncogenic routes affecting epigenetic landscape and tumour microenvironment. Mol. Oncol..

[99] Robinson D.J., Patel A., Purdie K.J., Wang J., Rizvi H., Hufbauer M., Ostano P., Akgül B., Chiorino G., Harwood C.A. (2019). Epigenetic Regulation of IASPP-P63 Feedback loop in cutaneous squamous cell carcinoma. J. Invest. Dermatol..

[100] Neilsen P.M., Noll J.E., Suetani R.J., Schulz R.B., Al-Ejeh F., Evdokiou A., Lane D.P., Callen D.F. (2011). Mutant p53 uses p63 as a molecular chaperone to alter gene expression and induce a pro-invasive secretome. Oncotarget..

[101] Shakya R., Tarulli G.A., Sheng L., Lokman N.A., Ricciardelli C., Pishas K.I., Selinger C.I., Kohonen-Corish M.R.J., Cooper W.A., Turner A.G. (2017). Mutant p53 upregulates alpha-1 antitrypsin expression and promotes invasion in lung cancer. Oncogene..

[102] Cerami E., Gao J., Dogrusoz U., Gross B.E., Sumer S.O., Aksoy B.A., Jacobsen A., Byrne C.J., Heuer M.L., Larsson E. (2012). The cbio cancer genomics portal: An open platform for exploring multidimensional cancer genomics data. Cancer Discov..

[103] Gao J., Aksoy B.A., Dogrusoz U., Dresdner G., Gross B., Sumer S.O., Sun Y., Jacobsen A., Sinha R., Larsson E. (2013). Integrative analysis of complex cancer genomics and clinical profiles using the cBioPortal. Sci Signal..

[104] Pfister N.T., Prives C. (2017). Transcriptional regulation by wild-type and cancer-related mutant forms of P53. Cold Spring Harb. Perspect. Med..

[105] Kim M.P., Lozano G. (2018). Mutant P53 Partners in crime. Cell Death Differ..

[106] Yue X., Zhao Y., Xu Y., Zheng M., Feng Z., Hu W. (2017). Mutant P53 in cancer: Accumulation, gain-of-function, and therapy. J. Mol. Biol..

[107] Rahnamoun H., Hong J., Sun Z., Lee J., Lu H., Lauberth S.M. (2018). Mutant P53 regulates enhancer-associated h3k4 monomethylation through interactions with the methyltransferase MLL4. J. Biol. Chem..

[108] Pfister N.T., Fomin V., Regunath K., Zhou J.Y., Zhou W., Silwal-Pandit L., Freed-Pastor W.A., Laptenko O., Neo S.P., Bargonetti J. (2015). Mutant P53 cooperates with the swi/snf chromatin remodeling complex to regulate VEGFR2 in breast cancer cells. Genes Dev..

[109] Zhu J., Sammons M.A., Donahue G., Dou Z., Vedadi M., Getlik M., Barsyte-Lovejoy D., Al-awar R., Katona B.W., Shilatifard A. (2015). Gain-of-Function P53 mutants co-opt chromatin pathways to drive cancer growth. Nature.

[110] Chitsazzadeh V., Coarfa C., Drummond J.A., Nguyen T., Joseph A., Chilukuri S., Charpiot E., Adelmann C.H., Ching G., Nguyen T.N. (2016). Cross-Species identification of genomic drivers of squamous cell carcinoma development across preneoplastic intermediates. Nat. Commun..

[111] Willis A., Jung E.J., Wakefield T., Chen X. (2004). Mutant p53 exerts a dominant negative effect by preventing wild-type p53 from binding to the promoter of its target genes. Oncogene.

[112] Song H., Hollstein M., Xu Y. (2007). P53 Gain-of-Function cancer mutants induce genetic instability by inactivating ATM. Nat. Cell Biol..

[113] Zhou G., Liu Z., Myers J.N. (2016). TP53 Mutations in head and neck squamous cell carcinoma and their impact on disease progression and treatment response. J. Cell. Biochem..

[114] Tanaka N., Zhao M., Tang L., Patel A.A., Xi Q., Van H.T., Takahashi H., Osman A.A., Zhang J., Wang J. (2018). Gain-of-Function mutant p53 promotes the oncogenic potential of head and neck squamous cell carcinoma cells by targeting the transcription factors FOXO3a and FOXM1. Oncogene.

[115] Adorno M., Cordenonsi M., Montagner M., Dupont S., Wong C., Hann B., Solari A., Bobisse S., Rondina M.B., Guzzardo V. (2009). A Mutant-P53/Smad complex opposes p63 to empower TGF-β-induced metastasis. Cell.

[116] Stindt M.H., Muller P.A.J., Ludwig R.L., Kehrloesser S., Dötsch V., Vousden K.H. (2015). Functional interplay between MDM2, P63/P73 and mutant P53. Oncogene.

[117] Li Z., Gonzalez C.L., Wang B., Zhang Y., Mejia O., Katsonis P., Lichtarge O., Myers J.N., El-Naggar A.K., Caulin C. (2016). Cdkn2a suppresses metastasis in squamous cell carcinomas induced by the gain-of-function mutant p53(R172H). J. Pathol..

[118] Torchia E.C., Caulin C., Acin S., Terzian T., Kubick B.J., Box N.F., Roop D.R. (2012). Myc, Aurora Kinase A, and Mutant P53(R172H) Co-operate in a mouse model of metastatic skin carcinoma. Oncogene.

[119] Caulin C., Nguyen T., Lang G.A., Goepfert T.M., Brinkley B.R., Cai W.-W., Lozano G., Roop D.R. (2007). An inducible mouse model for skin cancer reveals distinct roles for gain- and loss-of-function p53 mutations. J. Clin. Invest..

[120] Miller M., Shirole N., Tian R., Pal D., Sordella R. (2016). The Evolution of tp53 mutations: From loss-of-function to separation-of-function mutants. J. Cancer Biol. Res..

[121] Murnyák B., Hortobágyi T. (2016). Immunohistochemical correlates of tp53 somatic mutations in cancer. Oncotarget.

[122] Kemp C.J., Donehower L.A., Bradley A., Balmain A. (1993). Reduction of p53 gene dosage does not increase initiation or promotion but enhances malignant progression of chemically induced skin tumors. Cell.

[123] Li G., Tron V., Ho V. (1998). Induction of squamous cell carcinoma in p53-deficient mice after ultraviolet irradiation. J. Invest. Dermatol..

[124] Li G., Ho V.C., Berean K., Tron V.A. (1995). Ultraviolet radiation induction of squamous cell carcinomas in p53 transgenic mice. Cancer Res..

[125] Martínez-Cruz A.B., Santos M., Lara M.F., Segrelles C., Ruiz S., Moral M., Lorz C., García-Escudero R., Paramio J.M. (2008). Spontaneous squamous cell carcinoma induced by the somatic inactivation of retinoblastoma and trp53 tumor suppressors. Cancer Res..

[126] Savar A., Acin S., Gonzalez C.L., El-Sawy T., Mejia O., Li Z., Esmaeli B., Lacy-Hulbert A., El-Naggar A.K., McCarty J.H. (2015). Loss of Epithelial P53 and Av Integrin cooperate through akt to induce squamous cell carcinoma yet prevent remodeling of the tumor microenvironment. Oncogene.

[127] Vieler M., Sanyal S. (2018). P53 Isoforms and their implications in cancer. Cancers.

[128] Senturk S., Yao Z., Camiolo M., Stiles B., Rathod T., Walsh A.M., Nemajerova A., Lazzara M.J., Altorki N.K., Krainer A. (2014). P53Ψ Is a Transcriptionally inactive p53 isoform able to reprogram cells toward a metastatic-like state. Proc. Natl. Acad. Sci. USA.

[129] Shirole N.H., Pal D., Kastenhuber E.R., Senturk S., Boroda J., Pisterzi P., Miller M., Munoz G., Anderluh M., Ladanyi M. (2016). TP53 Exon-6 truncating mutations produce separation of function isoforms with pro-tumorigenic functions. Elife.

[130] Xue Y., San Luis B., Lane D.P. (2019). Intratumour heterogeneity of p53 expression; causes and consequences. J. Pathol..

[131] Heerfordt I.M., Nissen C.V., Poulsen T., Philipsen P.A., Wulf H.C. (2016). Thickness of actinic keratosis does not predict dysplasia severity or p53 Expression. Sci. Rep..

[132] Page A., Navarro M., Suarez-Cabrera C., Alameda J.P., Casanova M.L., Paramio J.M., Bravo A., Ramirez A. (2016). Protective role of p53 in skin cancer: Carcinogenesis studies in mice lacking epidermal p53. Oncotarget.

[133] Capaci V., Mantovani F., Del Sal G. (2020). Amplifying tumor-stroma communication: An emerging oncogenic function of mutant p53. Front. Oncol..

[134] Riihilä P., Nissinen L., Kähäri V.-M. (2021). Matrix metalloproteinases in keratinocyte carcinomas. Exp. Dermatol..

[135] Ala-aho R., Grénman R., Seth P., Kähäri V.-M. (2002). Adenoviral delivery of p53 gene suppresses expression of collagenase-3 (MMP-13) in squamous carcinoma cells. Oncogene.

[136] Ahonen M., Ala-Aho R., Baker A.H., George S.J., Grénman R., Saarialho-Kere U., Kähäri V.-M. (2002). Antitumor activity and bystander effect of adenovirally delivered tissue inhibitor of metalloproteinases-3. Mol. Ther..

[137] Hombach S., Kretz M. (2016). Non-Coding RNAs: Classification, biology and functioning. Adv. Exp. Med. Biol..

[138] Statello L., Guo C.-J., Chen L.-L., Huarte M. (2021). Gene regulation by long non-coding rnas and its biological functions. Nat. Rev. Mol. Cell Biol..

[139] Shi J., Zhang Y., Zhou T., Chen Q. (2019). TsRNAs: The swiss army knife for translational regulation. Trends Biochem. Sci..

[140] Slack F.J., Chinnaiyan A.M. (2019). The role of non-coding rnas in oncology. Cell.

[141] Bartel D.P. (2009). MicroRNAs: Target recognition and regulatory functions. Cell.

[142] Chen S., Thorne R.F., Zhang X.D., Wu M., Liu L. (2020). Non-Coding RNAs, guardians of the p53 galaxy. Semin. Cancer Biol..

[143] Lin T., Hou P.-F., Meng S., Chen F., Jiang T., Li M.-L., Shi M.-L., Liu J.-J., Zheng J.-N., Bai J. (2019). Emerging roles of p53 related lncrnas in cancer progression: A systematic review. Int. J. Biol. Sci..

[144] Di Agostino S. (2020). The Impact of Mutant P53 in the Non-Coding RNA world. Biomolecules.

[145] Pruszko M., Milano E., Forcato M., Donzelli S., Ganci F., Di Agostino S., De Panfilis S., Fazi F., Bates D.O., Bicciato S. (2017). The mutant p53-id4 complex controls VEGFA isoforms by recruiting lncrna MALAT1. EMBO Rep..

[146] Garofoli M., Volpicella M., Guida M., Porcelli L., Azzariti A. (2020). The Role of Non-Coding RNAs as Prognostic Factor, Predictor of Drug Response or Resistance and pharmacological targets, in the cutaneous squamous cell carcinoma. Cancers.

[147] Feng C., Zhang H.-L., Zeng A., Bai M., Wang X.-J. (2020). Tumor-suppressive microrna-216b binds to tpx2, activating the p53 signaling in human cutaneous squamous cell carcinoma. Mol. Ther. Nucleic Acids.

[148] Matson D.R., Denu R.A., Zasadil L.M., Burkard M.E., Weaver B.A., Flynn C., Stukenberg P.T. (2021). High nuclear tpx2 expression correlates with tp53 mutation and poor clinical behavior in a large breast cancer cohort, but is not an independent predictor of chromosomal instability. BMC Cancer.

[149] Geusau A., Borik-Heil L., Skalicky S., Mildner M., Grillari J., Hackl M., Sunder-Plassmann R. (2020). Dysregulation of tissue and serum micrornas in organ transplant recipients with cutaneous squamous cell carcinomas. Heal. Sci. Rep..

[150] García-Sancha N., Corchado-Cobos R., Pérez-Losada J., Cañueto J. (2019). MicroRNA dysregulation in cutaneous squamous cell carcinoma. Int. J. Mol. Sci..

[151] Piipponen M., Nissinen L., Riihilä P., Farshchian M., Kallajoki M., Peltonen J., Peltonen S., Kähäri V.-M. (2020). p53-regulated long noncoding RNA PRECSIT promotes progression of cutaneous squamous cell carcinoma via STAT3 signaling. Am. J. Pathol..

[152] Yang J., Adli M. (2019). Mapping and making sense of noncoding mutations in the genome. Cancer Res..

[153] Gao P., Wei G.-H. (2017). Genomic insight into the role of lncrna in cancer susceptibility. Int. J. Mol. Sci..

[154] Barbieri I., Kouzarides T. (2020). Role of RNA Modifications in Cancer. Nat. Rev. Cancer.

[155] Rowe D.E., Carroll R.J., Day C.L. (1992). Prognostic factors for local recurrence, metastasis, and survival rates in squamous cell carcinoma of the skin, ear, and lip. Implications for treatment modality selection. J. Am. Acad Derm..

[156] Johnson T.M., Rowe D.E., Nelson B.R., Swanson N.A. (1992). Squamous cell carcinoma of the skin (excluding lip and oral mucosa). J. Am. Acad. Derm..

[157] Stratigos A.J., Garbe C., Dessinioti C., Lebbe C., Bataille V., Bastholt L., Dreno B., Concetta Fargnoli M., Forsea A.M., Frenard C. (2020). European interdisciplinary guideline on invasive squamous cell carcinoma of the skin: Part 2. Treatment. Eur. J. Cancer.

[158] Carucci J.A., Martinez J.C., Zeitouni N.C., Christenson L., Coldiron B., Zweibel S., Otley C.C. (2004). In-transit metastasis from primary cutaneous squamous cell carcinoma in organ transplant recipients and nonimmunosuppressed patients: Clinical characteristics, management, and outcome in a series of 21 patients. Derm. Surg..

[159] Maubec E. (2020). Update of the management of cutaneous squamous-cell carcinoma. Acta Derm. Venereol..

[160] Migden M.R., Rischin D., Schmults C.D., Guminski A., Hauschild A., Lewis K.D., Chung C.H., Hernandez-Aya L., Lim A.M., Chang A.L.S. (2018). PD-1 blockade with cemiplimab in advanced cutaneous squamous-cell carcinoma. N. Engl. J. Med..

[161] Hillen U., Leiter U., Haase S., Kaufmann R., Becker J., Gutzmer R., Terheyden P., Krause-Bergmann A., Schulze H.J., Hassel J. (2018). Advanced cutaneous squamous cell carcinoma: A retrospective analysis of patient profiles and treatment patterns—Results of a non-interventional study of the DeCOG. Eur. J. Cancer..

[162] Stevenson M.L., Wang C.Q., Abikhair M., Roudiani N., Felsen D., Krueger J.G., Pavlick A.C., Carucci J.A. (2017). Expression of programmed cell death ligand in cutaneous squamous cell carcinoma and treatment of locally advanced disease with pembrolizumab. JAMA Dermatol..

[163] Slater N.A., Googe P.B. (2016). PD-L1 expression in cutaneous squamous cell carcinoma correlates with risk of metastasis. J. Cutan. Pathol..

[164] Grob J.-J., Gonzalez R., Basset-Seguin N., Vornicova O., Schachter J., Joshi A., Meyer N., Grange F., Piulats J.M., Bauman J.R. (2020). Pembrolizumab monotherapy for recurrent or metastatic cutaneous squamous cell carcinoma: A single-arm phase II trial (KEYNOTE-629). J. Clin. Oncol..

[165] Pugacheva E.N., Ivanov A.V., Kravchenko J.E., Kopnin B.P., Levine A.J., Chumakov P.M. (2002). Novel gain of function activity of p53 mutants: Activation of the dUTPase gene expression leading to resistance to 5-fluorouracil. Oncogene.

[166] Braun M.W., Iwakuma T. (2016). Regulation of cytotoxic T-cell responses by p53 in cancer. Transl. Cancer Res..

[167] Yoon K.W., Byun S., Kwon E., Hwang S.-Y., Chu K., Hiraki M., Jo S.-H., Weins A., Hakroush S., Cebulla A. (2015). Control of signaling-mediated clearance of apoptotic cells by the tumor suppressor p53. Science.

[168] Agupitan A.D., Neeson P., Williams S., Howitt J., Haupt S., Haupt Y. (2020). P53: A guardian of immunity becomes its saboteur through mutation. Int. J. Mol. Sci..

[169] Li L., Li M., Wang X. (2020). Cancer type-dependent correlations between TP53 mutations and antitumor immunity. DNA Repair.

[170] Sabapathy K., Lane D.P. (2017). Therapeutic targeting of p53: All mutants are equal, but some mutants are more equal than others. Nat. Rev. Clin. Oncol..

[171] Merino D., Kelly G.L., Lessene G., Wei A.H., Roberts A.W., Strasser A. (2018). BH3-mimetic drugs: Blazing the trail for new cancer medicines. Cancer Cell.

[172] Michel M., Kaps L., Maderer A., Galle P.R., Moehler M. (2021). The role of p53 dysfunction in colorectal cancer and its implication for therapy. Cancers.

[173] Zhu G., Pan C., Bei J.-X., Li B., Liang C., Xu Y., Fu X. (2020). Mutant p53 in cancer progression and targeted therapies. Front. Oncol..

[174] Timofeev O., Stiewe T. (2021). Rely on Each Other: Dna binding cooperativity shapes p53 functions in tumor suppression and cancer therapy. Cancers.

[175] Tang X., Zhu Y., Han L., Kim A.L., Kopelovich L., Bickers D.R., Athar M. (2007). CP-31398 restores mutant p53 tumor suppressor function and inhibits UVB-induced skin carcinogenesis in mice. J. Clin. Investig..

[176] https://www.clinicaltrials.gov/ct2/show/NCT02999893.

[177] Yu X., Vazquez A., Levine A.J., Carpizo D.R. (2012). Allele-specific p53 mutant reactivation. Cancer Cell..

[178] Weinmann L., Wischhusen J., Demma M.J., Naumann U., Roth P., DasMahapatra B., Weller M. (2008). A novel p53 rescue compound induces p53-dependent growth arrest and sensitises glioma cells to Apo2L/TRAIL-induced apoptosis. Cell Death Differ..

[179] https://clinicaltrials.gov/ct2/show/NCT02842125.

[180] https://clinicaltrials.gov/ct2/show/NCT03544723.

[181] https://clinicaltrials.gov/ct2/show/NCT00017173.

[182] https://clinicaltrials.gov/ct2/show/NCT00404339.

[183] https://clinicaltrials.gov/ct2/show/NCT02432963.

